# Pediatric Benign Paroxysmal Positional Vertigo: Degree of Nystagmus and Concurrent Dizziness Differs from Adult BPPV

**DOI:** 10.3390/jcm13071997

**Published:** 2024-03-29

**Authors:** Jun Beom An, Jisu Kim, Seok Hyun Park, Joonsung Yoon, Oak Sung Choo, Su-Kyoung Park, Jiwon Chang

**Affiliations:** 1 Department of Otorhinolaryngology-Head & Neck Surgery, Hallym University College of Medicine, Kangnam Sacred Heart Hospital, Seoul 07441, Republic of Korea; 2 Hallym Medical School, Hallym University College of Medicine, Chuncheon Campus, Chuncheon 24252, Republic of Korea; 3 PSK99-Ear Nose and Throat Clinic, Seoul 07442, Republic of Korea; 4 Department of Otorhinolaryngology-Head & Neck Surgery, Korea University Anam Hospital, 73 Goryeodae-ro, Seongbuk-gu, Seoul 02841, Republic of Korea

**Keywords:** benign paroxysmal positional vertigo (BPPV), pediatric, dizziness, vertigo, nystagmus, concurrent disease, recurrence

## Abstract

**Background**: Benign paroxysmal positional vertigo (BPPV) is characterized by brief, intense episodes of vertigo triggered by abrupt changes in head position. It is generally accepted as being most common in adults, while it is regarded as rare in children. It is necessary to compare the disease between pediatric and adult patients for a better understanding of the disease’s characteristics and its natural history. This study aimed to identify the clinical characteristics of BPPV in children and compare them with those of adult BPPV patients. **Methods**: All children ≤ 18 years old who were diagnosed with BPPV were selected by searching the electronic database of our hospital. Clinical features were identified by medical record review. For adult patients, we collected data from patients > 19 years of age. **Results**: A total of 30 pediatric (13.65 ± 4.15 years old) and 264 adult patients (60.86 ± 13.74 years old) were included in the study. Among pediatric patients, the lateral canals were involved in 80% and the posterior canals in 16.67%. In adult patients, the lateral and posterior canals were involved similarly (*p* = 0.007). The degree of nystagmus in pediatric patients was 6.82 ± 12.09, while in adults it was 15.58 ± 20.90 (*p* < 0.001). The concurrent dizziness disorder was higher in the pediatric group and recurrence was higher in the adult group. In the regression analysis, it was found that adult patients had a stronger nystagmus with a value of 6.206 deg/sec, and the risk of concurrent dizziness disorder was found to be 5.413 times higher in the pediatric group (*p* < 0.05). **Conclusions**: BPPV occurs in pediatric patients with lower prevalence, but it cannot be overlooked. In the pediatric group, a relatively high proportion of patients demonstrated lateral canal involvement, weaker nystagmus, and additional dizziness disorder.

## 1. Introduction

Benign paroxysmal positional vertigo (BPPV) is characterized by brief, intense episodes of vertigo triggered by abrupt changes in head position. BPPV is diagnosed when typical nystagmus and dizziness symptoms occur following positional tests. It is a prevalent vestibular disorder in adults, accounting for 17–42% of patients with vertigo [1,2]. The prevalence of the disease is increased among elderly persons and women, with a peak onset between 50 and 60 years of age and a female-to-male ratio of 2:1 to 3:1 [3]. While BPPV is well studied in adults, pediatric BPPV presents a unique set of challenges due to the rarity of the disease in pediatric patients.

Vertigo and dizziness occur less frequently in children compared to adults. The prevalence of vestibular disorders ranges between 0.7% and 15% [4], and children differ from adults in the causes of dizziness. According to several studies, the most common diseases that occur in pediatric dizzy patients are vestibular migraine of childhood (VMC), recurrent vestibulopathy of childhood (RVC), and orthostatic dizziness [5,6]. Also, the causes of pediatric dizziness vary according to age category; RVC and VMC are most common in the preschool age group, while RVC, VMC, and psychogenic vertigo are common in the school-age group, and VMC, orthostatic dizziness are common in the adolescent group [5,7].

As one of the mechanisms explaining BPPV is age-related atrophy of the utricle, BPPV is generally accepted as being most common in adults, while it is regarded as a rare entity in children [4,5,7]. BPPV of the posterior semicircular canal (PSCC) in pediatric patients was first reported in 1987 [8], and lateral semicircular canal (LSCC) BPPV was reported in 2003 [9]. Several studies have reported the characteristics of pediatric BPPV such as causes, prevalence, demographics, involved canals, complications, and responses to treatment [10,11,12]; however, since BPPV is caused by abrupt detachment and movement of otoliths—which is presumed to be due to degenerative changes in the utricle—it is necessary to compare the disease between pediatric patients and adult patients for a better understanding of the disease characteristics and its natural history. It has been recently noted that pediatric patients with dizziness may have simultaneous coexisting disorders, which are not typically seen in adult populations [10,11]. Therefore, BPPV in children might have been previously overlooked. In this study, we compared and analyzed the clinical features of pediatric and adult BPPV patients. This study aimed to identify the clinical characteristics of BPPV in children and compare them with those of adult BPPV patients.

## 2. Materials and Methods

We conducted a retrospective review of pediatric patients ≤18 years of age presenting with dizziness or vertigo at our university hospital between January 2014 and December 2023. Patients who received the positional test for diagnosis and the repositioning maneuver for treatment in these periods were collected by searching the electronic database of our hospital. Further review of individual medical records was conducted to identify clinical features including demographics, degree of nystagmus, concurrent dizziness disorder, comorbidities, canal involvement, multicanal involvement, canal conversion, frequency of repositioning methods, and incidence of recurrence. For adult patients, we collected data from patients ≥19 years of age who received the positional test for diagnosis and the repositioning maneuver for treatment between January 2022 and December 2022. Further review of individual medical records was conducted to identify clinical features as mentioned above. This study was approved by the Institutional Review Board at the hospital where the study was conducted (HKS 2018-03-006-002).

All patients underwent a comprehensive evaluation that included taking a detailed history as well as otological and neurological examinations by experts in our Otolaryngology departments. Consultations were referred to the Pediatric Department for pediatric patients and to the Neurology Department for adult patients when there was a need to rule out other diseases. All patients underwent positional tests using videonystagmography (ICS chartr 200) goggles in the following order: spontaneous, bow test, lean test, right/left Dix–Hallpike test, and right/left supine head roll test. BPPV was diagnosed when vertigo and characteristic nystagmus were present with the diagnostic maneuver. The Epley maneuver was used to treat PSCC, the barbecue maneuver or Kim’s maneuver was used to treat LSCC, and the reverse Epley maneuver or the Yacovino maneuver were used to treat ASCC. The maneuvers were repeated if symptoms were present at the next follow-up.

BPPV canals were identified by the involved canals, sides, and types (canalolithiasis or cupulolilthiasis). The degree of nystagmus was defined as the degree of maximal SEV (slow eye velocity) during the positional test. Resolution of BPPV was defined as the resolution of symptoms along with the absence of nystagmus on follow-up positional tests. Patients were considered to have multicanal BPPV if the simultaneous involvement of more than one canal was observed. Canal conversion was defined if a different canal was involved at the next follow-up positional test. Patients were considered to have recurrence if the patient revisited with a new episode of BPPV at least 3 months after previous episodes of BPPV.

The concurrent dizziness disorder was diagnosed according to various diagnostic criteria. Comorbid diseases included diabetes, hypertension, dyslipidemia, cerebral vascular disease, cardiovascular disease, thyroid disease, and immune-related diseases.

Characteristics between the two groups were evaluated using Fisher’s exact test for categorical variables and a Mann–Whitney test for continuous variables. Characteristics between the different age groups were evaluated using the Kruskal–Wallis test for multiple comparisons. Multiple regression analyses were performed to identify the difference in the degree of nystagmus between adult and pediatric patients and a logistic regression analysis was performed to validate the difference in concurrent dizziness disorder between the two groups. A *p*-value of <0.05 was considered to be statistically significant. Statistical analyses were conducted using SPSS v.22.0 (IBM, Armonk, NY, USA).

## 3. Results

A total of 30 pediatric patients and 264 adult patients were enrolled in the study. In the pediatric group (4–18 years old), the mean age was 13.60 ± 4.15, and the median age was 14.5 (ranging from 5 to 18) (Table 1). For the adult group (19 years and older), the mean age was 60.86 ± 13.74, and the median age was 63 (ranging from 22 to 90). While there was no significant sex difference in the pediatric group, the adult group exhibited a higher prevalence of the condition in females compared to males. In the pediatric group, 5 patients were admitted while 25 patients were treated at the outpatient clinic; all adult patients were managed in the outpatient clinic. The mean observation period (weeks) for the pediatric group was 7.03 ± 13.25 weeks, with a median of 2.5 weeks (ranging from 0 to 60). In the adult group, the mean observation period was 4.23 ± 7.32 weeks, and the median was 2 weeks (ranging from 0 to 74).

**Figure 1:**
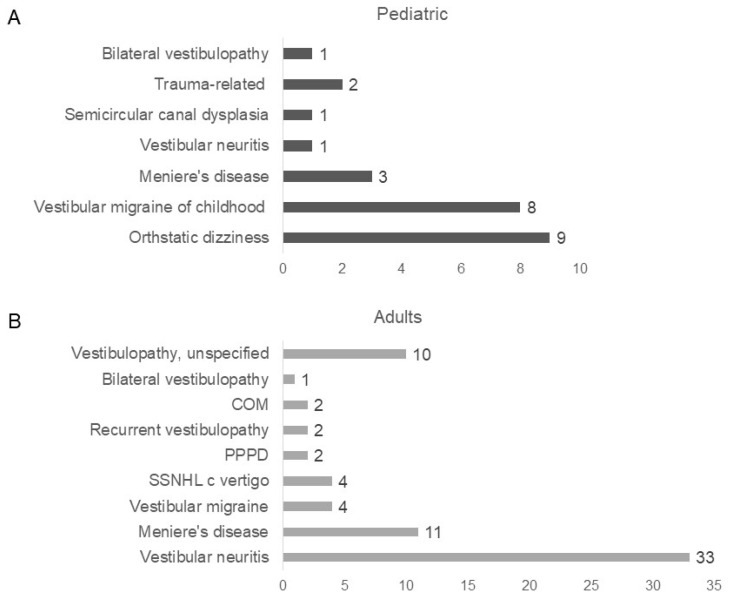
Concurrent dizziness disorder in pediatric and adult patients: (**A**) In pediatric patients, 19 patients had concurrent dizziness disorder. Nine patients had orthostatic dizziness (including orthostatic hypotension, postural orthostatic tachycardiac syndrome), 8 had VMC, 3 had Meniere’s disorder, 1 had vestibular neuritis, 1 had semicircular canal dysplasia, 2 had trauma-related disorder, and 1 had bilateral vestibulopathy; (**B**) In adult patients, 69 patients had concurrent dizziness disorder. Thirty-three patients had vestibular neuritis, 11 had Meniere’s disease, 4 had vestibular migraine, 4 had sudden sensorineural hearing loss with vertigo, 2 had PPPD, 2 had recurrent vestibulopathy, 2 had chronic otitis media, 1 had bilateral vestibulopathy, and 10 had vestibulopathy unspecified.

Concurrent dizziness disorder was higher in the pediatric group, with 19 cases (63.33%) compared to 69 cases (26.14%) in adults (Table 1). In pediatric patients, orthostatic dizziness including orthostatic hypotension, postural orthostatic tachycardiac syndrome (POTS), and VMC were most often identified with BPPV (Figure 1A). Besides orthostatic dizziness and VMC, three patients had Meniere’s disease, two had trauma-related vestibular disorders, one had vestibular neuritis, one had bilateral vestibulopathy, and one had semicircular canal dysplasia. In adult patients, vestibular neuritis and Meniere’s disease were most often accompanied by BPPV (Figure 1B). Meanwhile, comorbid diseases were lower in the pediatric group, with 1 case (3.33%) compared to 120 cases (45.45%) in adults (Table 1).

In pediatric BPPV cases, lateral canal involvement was observed in 24 cases (80%), while posterior canal involvement was seen in 5 cases (16.67%), indicating a higher prevalence of lateral canal involvement (Table 2); however, in adult cases, lateral canal involvement was observed in 131 cases (49.62%), and posterior canal involvement was observed in 123 cases (46.59%), showing a similar distribution between the two canal types (*p* = 0.007). There were no significant differences in the right or left side preferences between both pediatric and adult cases. In pediatric patients, both canalolithiasis and cupulolithiasis showed similar distribution, with 18 cases (60.00%) and 12 cases (40.00%), respectively; however, among adult patients, 209 cases (79.17%) were classified as canalolithiasis and 52 cases (19.70%) as cupulolithiasis.

**Figure 2:**
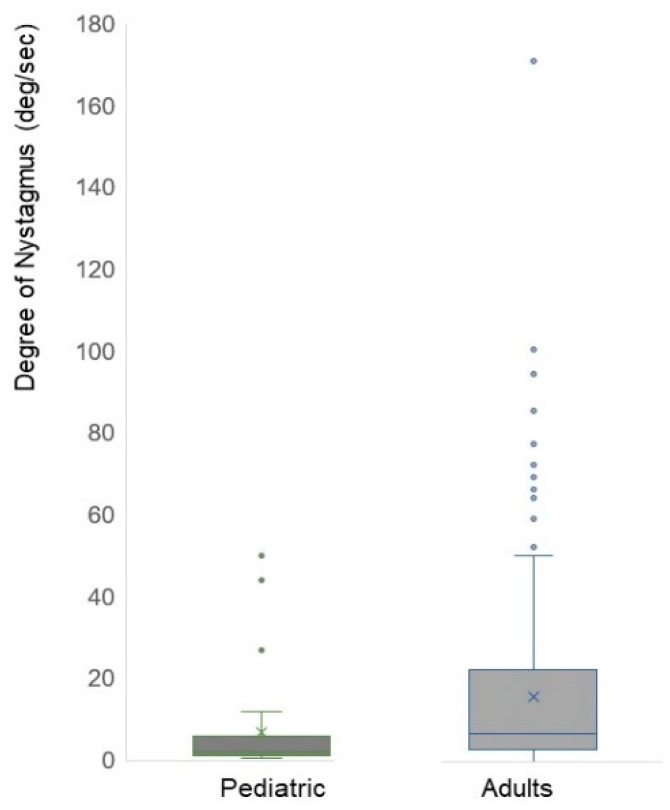
Degree of nystagmus in pediatric and adult patients. The mean degree of nystagmus in pediatric patients was 6.82 ± 12.09 deg/s, whereas adult patients showed a mean degree of 15.58 ± 20.90 deg/s, demonstrating a statistically significant difference (*p* < 0.001). Among the 30 pediatric patients, three patients exhibited a stronger degree of nystagmus than others with values of 44, 94, and 50, respectively; however, in most pediatric patients, nystagmus degrees were measured at 9 or below. Dots are the value of nystagmus degree per each patient.

The mean degree of nystagmus in pediatric patients was 6.82 ± 12.09 deg/s, whereas adult patients showed a mean degree of 15.58 ± 20.90 deg/s, demonstrating a statistically significant difference (*p* < 0.001) (Table 2). Among the thirty pediatric cases, three cases exhibited higher nystagmus sizes than others with values of 44, 94, and 50, respectively; however, in most pediatric patients, nystagmus degrees were measured at 9 or below (Figure 2).

Among the 264 adult patients analyzed for BPPV, there were 79 males and 185 females (Table 3). When the age groups of adults were divided into 20–40 years, 41–60 years, and 61 years and above, the number of patients increased with age. In males, there were 7 (8.86%), 24 (30.38%), and 48 (60.76%) in each age group, respectively. In females, the number of patients increased with age: 16 (8.65%) in the youngest group, 65 (35.14%) in the middle group, and 104 (56.22%) in the oldest group. When we compared pediatric patients to adults in different age groups (20–40 vs. 41–60 vs. ≥61), coexisting dizziness disorder was higher in pediatric patients (19 cases, 63.33%) compared to adults (8 cases (34.78%) in the 20–40 age group; with 18 cases (20.22%) in the 41–60 age group; and 43 cases (28.29%) in the ≥61 age group).

The degree of nystagmus in pediatric patients was significantly lower when compared to adults across different age groups (Table 4). The recurrence rate of BPPV was observed to be 2 cases (6.67%) in pediatric patients, 4 cases (17.39%) in the 20–40 age group, 17 cases (19.10%) in the 41–60 age group, and 37 cases (24.34%) in ≥61 age group, showing an increasing trend with age although it was not statistically significantly (*p* = 0.162); however, recurrence was significantly higher in the ≥61 age group than in pediatric patients (*p* = 0.030).

In the regression analysis, adult patients showed a stronger intensity of nystagmus with a value of 6.206 deg/sec, although it was not statistically significant (*p* = 0.287) (Table 5). The possibility of concurrent dizziness disorder was 5.413 times higher in pediatric patients with statistical significance (*p* < 0.05) (Table 6).

## 4. Discussion

In our study, we selected 30 pediatric and 264 adult BPPV patients who performed the positional test and repositioning maneuver by searching the electronic database of our hospital. The mean age was 13.60 ± 4.15 and 60.86 ± 13.74 for each group, and the male-to-female ratios were 1:1.14 and 1:2.34, respectively. We identified several different characteristics of BPPV between pediatric and adult patients. The lateral canal was more commonly affected in pediatric patients, while the posterior and lateral canals were similarly affected in the adult group. Adult patients had more canalolithiasis type than cupulolithiasis, and the degree of nystagmus was weaker in pediatric patients. Concurrent dizziness disorder was more frequent in the pediatric group, and recurrence was higher in the adult group.

BPPV was relatively rare in pediatric patients, but in our study it was also rare in the adult age group of 20–40 years old. The prevalence almost increased 3.9-fold in the age group of 40–60 years old, and 6.6-fold in the age group of ≥60 years old. Consistent with the previous reports that the age onset of BPPV is between the fifth and seventh decades of life [8], our data also support that BPPV might be related to the degenerative change in the vestibule. However, a female-to-male ratio was distinct between the pediatric and the adult patients. There was no difference in the prevalence of BPPV in pediatric patients but females were more affected by BPPV with a ratio of 2.17–2.71 in adults over 20 years old. Several studies have shown that the ratio of females affected by BPPV is higher than males, ranging from 2.2:1.5 to 1 [13]. Hormonal fluctuations in females, including a decrease in estrogen levels, can disrupt the metabolism of otoconia and reduce the expression of otoconial components and anchoring proteins, which increases the likelihood of developing BPPV [14,15,16].

In our study, the lateral semicircular canal was primarily affected in pediatric patients (80%) while both lateral and posterior canals were similarly involved in adult patients. Some studies have reported a higher involvement of the posterior canal compared to the lateral canal in pediatric patients [10,11], and mentioned an increased prevalence of superior and lateral canal involvement relative to adults [10]. Others reported similar involvement of both canals [12] in pediatric patients. There is an inconsistency between studies but pediatric patients likely present with involvement of the lateral canal more often than adult patients. This might be due to the change in the angular orientation of the lateral canal according to age group [17] or due to sports activities inducing various head positions in pediatric patients [10]. Also, in our study, a relatively high proportion of adult patients presented with lateral canal involvement. This is probably due to the easy accessibility of hospitals and the medical service system in Korea. Patients with BPPV can visit the clinic within 1–7 days of symptom onset, and lateral canal BPPV tends to spontaneously resolve more often than posterior canal BPPV. Although the distribution of canalolithiasis or cupulolithiasis was similar between pediatric patients, the results were statistically significant (*p* = 0.056) in our study. Since the number of patients is insufficient to support the hypothesis, this finding may explain the degeneration of the utricle and the development of BPPV with increasing age.

The severity of the nystagmus was weaker in pediatric patients in our study (*p* < 0.001). Although it was not supported by statistics in the multiple regression analysis (*p* = 0.287), pediatric patients showed an average of 6.206 deg/s weaker nystagmus than adult patients. Out of the thirty pediatric patients, three patients aged 18, 17, and 18, respectively, showed higher nystagmus sizes of 44, 94, and 50. For most of the pediatric patients, nystagmus degrees were measured at 9 or below. Accordingly, it may be difficult to recognize and diagnose BPPV in pediatric patients due to a weaker degree of nystagmus, which can be overlooked. There is limited research on the relationship between the severity of symptoms and the degree of nystagmus, as well as the correlation between the amount of nystagmus and its severity. However, studies are reporting the morphology of otoconia in different age groups [18,19]. It is reported that most otoconial bodies are pitted, fissured, or broken into several fragments, and linking filaments are shown to be weakened or broken in the aged group. It seems that the fissured and broken otoconial bodies enter the semicircular canal more easily, which might induce severe nystagmus in adult patients but this should be studied further in the future.

In children, BPPV is reported to occur as a secondary condition concurrently with any vestibular disorders [11,20]. Therefore, physicians may consider other vestibular conditions as a primary diagnosis and not evaluate BPPV as a contributing factor for symptoms. In our study, 63.3% of pediatric patients had concurrent dizziness disorders. Unlike previous studies that report concussion, acute vestibular syndrome, or migraine as concurrent symptoms [10,21,22], we identified that quite a large proportion of pediatric patients had concurrent orthostatic dizziness. Considering that most of these orthostatic dizziness patients were diagnosed by changes in blood pressure or heart rate in the tilt table test, physicians should also pay attention while taking their history to whether pediatric patients complain of dizziness while changing to the upright position or lying/bending down. The relationship between orthostatic dizziness and BPPV is unclear but recent studies have shown the correlation between BPPV-induced otolith organ hypofunction and transient orthostatic hypotension [23,24].

The limitation of our study is that we recruited pediatric patients who have visited our clinic for the past 10 years, while we enrolled adult patients who have visited for 1 year. This was because of the rarity of the disease among pediatric patients. However, there were two otologic specialists constantly during the period when pediatric and adult patients visited. Also, although adult patients who visited our clinic during the specific 1 year were included in the study, we reviewed their medical records before and after that specific year in detail, which enabled us to obtain their data sufficiently.

## 5. Conclusions

BPPV occurs in pediatric patients with lower prevalence but it cannot be overlooked. In the pediatric BPPV, a relatively high proportion of patients demonstrated lateral canal involvement, weaker nystagmus, and additional concurrent dizziness disorder.

## Figures and Tables

**Table 1:** Demographics and clinical features of patients with BPPV (pediatric vs. adult).

Characteristics		Total Participants	
		Pediatric	Adult
N		30	264
Age at visit			
	Mean (SD)	13.60 (4.15)	60.86 (13.74)
	Median (range)	14.5 (5~18)	63 (22~90)
Sex, n (%)			
	Male	14 (46.67)	79 (29.92)
	Female	16 (53.33)	185 (70.08)
	Female-to-Male ratio	1.14	2.34
Duration of f/up (week)			
	Mean (SD)	7.03 (13.25)	4.23 (7.32)
	Median (range)	2.5 (0~60)	2 (0~74)
Concurrent dizziness disorder, n (%)		19 (63.33)	69 (26.14)
Comorbid disease (excluding dizziness), n (%)		1 (3.33)	120 (45.45)

**Table 2:** Presentations and outcomes of BPPV (pediatric vs. adult).

Characteristics		Total Participants		
		Pediatric	Adult	*p*-Value
Affected canal, n (%)				0.007 *
	posterior	5 (16.67)	123 (46.59)	
	lateral	24 (80.00)	131 (49.62)	
	superior	0 (0.00)	5 (1.89)	
Affected side, n (%)				0.190
	right	18 (60.00)	154 (58.33)	
	left	10 (33.33)	105 (39.77)	
	both	2 (6.67)	5 (1.89)	
Type, n (%)				0.056
	canalolithiasis	18 (60.00)	209 (79.17)	
	cupulolithiasis	12 (40.00)	52 (19.70)	
	both	0 (0.00)	3 (1.14)	
Nyst (deg/sec), mean (SD)		6.82 (12.09)	15.58 (20.90)	<0.001 *
Multicanal, n (%)		3 (10.00)	27 (10.23)	1.000
Number of CRP, mean (SD)		1.57 (0.94)	1.88 (1.94)	0.733
Recurrent, n (%)		2 (6.67)	58 (21.97)	0.055
Recurrent number, mean (SD)		1.00 (0.00)	1.96 (1.16)	0.181
Canal conversion, n (%)		0 (0.00)	21 (7.95)	0.145

* Mann–Whitney test or Fisher’s exact test, significance at *p* < 0.05.

**Table 3:** Demographics and clinical features of patients with BPPV in different age groups (pediatric vs. 20–40 vs. 41–60 vs. 61+).

Characteristics		Pediatric	Adults		
			20–40	41–60	61+
N		30	23	89	152
Sex, n (%)					
	Male	14 (46.67)	7 (30.43)	24 (26.97)	48 (31.58)
	Female	16 (53.33)	16 (69.57)	65 (73.03)	104 (68.42)
	Female-to-Male ratio	1.14	2.29	2.71	2.17
Duration of f/up (week)					
	Mean (SD)	7.03 (13.25)	5.65 (12.45)	4.02 (5.40)	4.14 (7.31)
	Median (range)	2.50 (0~60)	2 (0~60)	1 (0~28)	2 (0~74)
Concurrent dizziness disorder, n (%)		19 (63.33)	8 (34.78)	18 (20.22)	43 (28.29)
Comorbid disease (excluding dizziness), n (%)		1 (3.33)	2 (8.70)	30 (33.71)	88 (57.89)

**Table 4:** Presentations and outcomes of BPPV in different age groups (pediatric vs. 20–40 vs. 41–60 vs. 61+).

Characteristics			Adults			
		Pediatric	20–40	41–60	61+	*p*-Value
Affected canal, n (%)						0.096
	posterior	5 (16.67)	13 (56.52)	42 (47.19)	68 (44.74)	
	horizontal	24 (80.00)	10 (43.48)	43 (48.31)	78 (51.32)	
	superior	0 (0.00)	0 (0.00)	2 (2.25)	3 (1.97)	
Affected side, n (%)						0.417
	right	18 (60.00)	13 (56.52)	47 (52.81)	94 (61.84)	
	left	10 (33.33)	10 (43.48)	41 (46.07)	54 (35.53)	
	both	2 (6.67)	0 (0.00)	1 (1.12)	4 (2.63)	
Type, n (%)						0.154
	canalolithiasis	18 (60.00)	18 (78.26)	74 (83.15)	117 (76.97)	
	cupulolithiasis	12 (40.00)	5 (21.74)	15 (16.85)	32 (21.05)	
	both	0 (0.00)	0 (0.00)	0 (0.00)	3 (1.97)	
Nyst (deg/sec), mean (SD)		6.82 (12.09)	15.96 (35.05)	13.52 (18.89)	16.82 (19.53)	<0.001 *
Multicanal, n (%)		3 (10.00)	4 (17.39)	7 (7.87)	16 (10.53)	0.560
Number of CRP, mean (SD)		1.57 (0.94)	1.65 (0.98)	1.88 (1.70)	1.91 (2.18)	0.985
Recurrent, n (%)		2 (6.67) ^†^	4 (17.39)	17 (19.10)	37 (24.34) ^†^	0.162
Recurrent number, mean (SD)		1.00 (0.00)	1.75 (0.96)	1.93 (0.88)	2.00 (1.29)	0.591
Canal conversion, n (%)		0 (0.00)	2 (8.70)	9 (10.11)	10 (6.58)	0.286

* Kruskal–Wallis test, significance at *p* < 0.05. ^†^ Recurrence was significantly higher in the ≥61 age group than in pediatric patients (*p* = 0.030).

**Table 5:** Multiple regression analysis for the degree of nystagmus between adult and pediatric patients.

Parameter	Estimate	Standard Error	t-Value	Pr > |t|
Adult (vs. Pediatric)	6.206	5.816	1.07	0.287

**Table 6:** Logistic regression analysis for the concurrent dizziness between adult and pediatric patients.

Effect	Point Estimate	95% Wald	Confidence Limits	Pr > ChiSq
Pediatric (vs. Adult)	5.413	1.559	18.793	0.008

## Data Availability

All the data are available in the manuscript.
